# Are HIV-1-Specific Antibody Levels Potentially Useful Laboratory Markers to Estimate HIV Reservoir Size? A Review

**DOI:** 10.3389/fimmu.2021.786341

**Published:** 2021-11-11

**Authors:** Silvere D. Zaongo, Feng Sun, Yaokai Chen

**Affiliations:** Division of Infectious Diseases, Chongqing Public Health Medical Center, Chongqing, China

**Keywords:** HIV-1-specific antibody, marker, HIV-1 DNA, HIV reservoir, Level

## Abstract

Despite the benefits achieved by the widespread availability of modern antiretroviral therapy (ART), HIV RNA integration into the host cell genome is responsible for the creation of latent HIV reservoirs, and represents a significant impediment to completely eliminating HIV infection in a patient *via* modern ART alone. Several methods to measure HIV reservoir size exist; however, simpler, cheaper, and faster tools are required in the quest for total HIV cure. Over the past few years, measurement of HIV-specific antibodies has evolved into a promising option for measuring HIV reservoir size, as they can be measured *via* simple, well-known techniques such as the western blot and enzyme-linked immunosorbent assay (ELISA). In this article, we re-visit the dynamic evolution of HIV-1-specific antibodies and the factors that may influence their levels in the circulation of HIV-positive individuals. Then, we describe the currently-known relationship between HIV-1-specific antibodies and HIV reservoir size based on study of data from contemporary literature published during the past 5 years. We conclude by highlighting current trends, and discussing the individual HIV-specific antibody that is likely to be the most reliable antibody for potential future utilization for quantification of HIV reservoir size.

## Introduction

Today, the human immunodeficiency virus (HIV) infection remains a major public health burden despite four decades of massive monetary and intellectual investment into HIV research globally (1). Since 2010, the proportion of HIV-1 infected individuals receiving ART has increased. For example, in 2020, 27.4 million of the 37.6 million people living with HIV (PLWH) are reported to be on ART, which is more than triple the number of patients on ART recorded in 2010 (7.8 million). It is estimated that since 2001, the use of modern ART has prevented 16.2 million deaths. The preceding report also indicates that the number of AIDS-related deaths has fallen by 43% between 2010 and 2020 (2). Indeed, modern antiretroviral therapy (ART) efficiently suppresses HIV-1 replication by targeting key mechanisms in its life cycle (3), which in turn (i) reduces HIV viral RNA load to below detectable levels (4, 5), (ii) increases the number of CD4+ T-cells (6, 7), (iii) reduces the incidence of AIDS-related diseases and/or deaths (6, 8), and (iv) effectively prevents the transmission of HIV to uninfected people (9). However, despite the critically important impact of modern ART regimens, ART does not eliminate the virus from infected patients (10).

The insertion of a DNA copy of the HIV viral genome into the host cell chromosome is a critically important step of the life cycle of HIV. HIV subsequently hijacks the host cellular machinery to its advantage through viral protein and RNA production. An infected cell harboring HIV DNA remains infected for the life of that cell. Thus, ART only suppresses viral replication, and the cessation of ART use in the absence of HIV DNA elimination will inevitably be followed by viral rebound (11). HIV DNA integration results in the establishment of latent infection, leading to the creation of a latent HIV reservoir. Defined as quiescent host cells carrying an integrated copy of the viral genome that does not express HIV viral transcripts or proteins, the latent reservoir is the major component of the HIV reservoir; a minor component of the latent reservoir being the HIV active reservoir (12). The greatest challenge to HIV eradication is the persistence of latent HIV provirus in reservoir cells. Several mechanisms, described in past publications (13–15), are responsible for this outcome. In addition, researchers have observed that individuals harboring low HIV reservoir levels are able to control HIV replication in the absence of ART (16, 17). Therefore, the study of tools that are able to accurately measure HIV reservoir size is crucial for the monitoring of remission and/or prognosis of HIV-infected individuals (10).

Currently, several approaches to quantify the HIV reservoir exist, despite four inherent challenges. Firstly, the majority of proviruses persisting in people living with HIV taking ART harbor mutations and/or deletions that render these particular proviruses defective, and unable to replicate. Secondly, not all proviruses are able to produce viable virions after activation. Thirdly, the frequency of latently-infected cells is inherently very low. Finally, a large proportion of the HIV reservoir is present in tissues that cannot be accurately sampled using currently used specimen-collection approaches (18–21). The current methods used to quantify the HIV reservoir can be classified into four major groups, based on specific aspects of the HIV provirus and its functionality. Thus, there are currently assays (i) measuring levels of replication-competent virus or intact HIV genomes [the quantitative viral outgrowth assay (QVOA) (22, 23) and several PCR-based assays (24, 25)], (ii) measuring levels of translationally competent virus [the enhanced digital p24 single-molecule assay (SIMOA) (26–29), for example], (iii) measuring levels of transcriptionally competent virus [the qPCR and droplet digital PCR assays (30, 31)], and (iv) measuring total and integrated levels of HIV DNA (PCR quantification of total, integrated, and episomal HIV DNA) (12). To measure HIV reservoirs, researchers use biopsies from gut-associated lymphoid tissue (GALT) or lymph nodes (LN), or peripheral blood mononuclear cells (PBMCs) extracted either from 10 ml of blood, or *via* leukapheresis. It must be noted that none of these collected specimens, taken individually, provide a comprehensive picture of reservoir dynamics in patients taking ART. Moreover, commonly used methods regarded as “standard” (12), such as QVOA and its derivatives, are expensive, labor-intensive, require a large numbers of cells (~20-50 million CD4^+^ T-cells), require biosafety-containment, and have a tendency to underestimate the size of the replication-competent viral reservoir (18, 32). This highlights the urgent need for simpler, less expensive, and time-saving methods to reliably measure HIV reservoir size.

Of late, the estimation of HIV reservoir size using biomarkers, especially those biomarkers emanating from immune responses, has been recommended as a potentially realistic solution to the current difficulties related to accurate estimation of reservoir size. Thus, the utilization of antibodies for HIV-1 DNA profiling is likely to be simpler, less expensive, and results may be obtained more rapidly. Indeed, quantitative detection of HIV-1-specific antibodies is commonly used in-clinic, mainly *via* western blot (33, 34), enzyme-linked immunosorbent assay (ELISA) (35), laser induced plasma spectroscopy (LIPS) (36, 37), and microsphere-based array assay (38). Moreover, antibodies as biomarkers could possibly prove to be a significant means to overcome the four inherent challenges referred to in the preceding paragraph, which are encountered by existing approaches to quantify the HIV reservoir. Antibody production is not, to our knowledge, influenced by specific aspects of the HIV provirus and its functionality. Therefore, HIV-1-specific antibodies that are able to accurately predict HIV-1 reservoir size could represent an important and simpler option when considering curative HIV strategies (39, 40). Herein, we review the dynamic evolution of HIV-specific antibodies and the factors influencing their production. We also discuss the possibility of using HIV-1-specific antibodies to estimate HIV reservoir size based on investigational observations published within the past five years.

## The Evolution of HIV-1-Specific Antibodies

### HIV-1-Specific Antibodies Before ART Initiation

**Figure 1:**
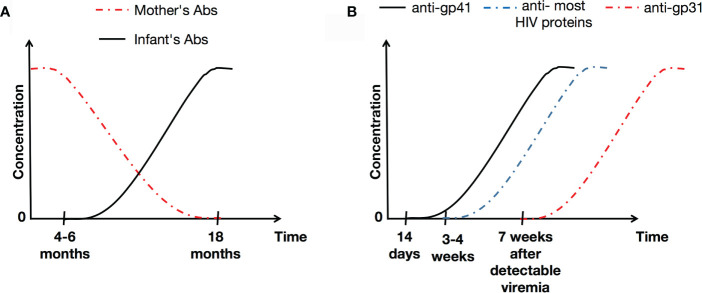
Illustration of HIV-1-specific antibody concentrations over the time in infants **(A)** and the general population **(B)**. Abs, antibodies.

An infant born to an HIV-positive mother acquires maternal antibodies *via* the placenta. The infant is HIV seropositive due to the passive transfer of maternal immunoglobulin (Ig) G antibodies, which occurs during the last trimester of pregnancy (41, 42). Some reports suggest that these IgG antibodies may persist for up to 18 months (43, 44). At 4–6 months of age, infants start producing their own IgG antibodies (against a limited number of HIV antigens) (45), characterized at first by production of anti-glycoprotein (gp) 160, and subsequently followed by anti-gp120 and anti-gp41 (46) (see **Figure 1A**).

In adults, it has been reported that the initial specific antibody response to HIV is detected 14 days after infection, and targets gp41 (47). In the absence of appropriate treatment, levels of gp41 remain stable over the time, and correlates with viral load (48). Generally, antibodies to most HIV proteins are detectable within 3-4 weeks of infection, although anti-p31 takes much longer to be produced (at around 7 weeks after detectable viremia) (49) (**Figure 1B**). It is known that in untreated HIV-positive adults, levels of antibodies to HIV are stable, and correlate positively with viral loads (48).

### HIV-1-Specific Antibodies After ART, and Duration on ART

During HIV infection, the administration of ART induces HIV-specific antibody levels to decline (48). Actually, antibodies of all types (with the exception of anti-p17) may be cleared in infants who initiate ART by 3 months of age as a direct consequence of the rapid control of HIV-1 replication (50). It has also been observed that anti-gp41 levels decrease with duration of ART (Pearson r=-026, *p*<0.0001) (51). In addition, it has been demonstrated (52) that infants on ART with effective viral suppression (<400 RNA copies/mL) have (i) low but stable levels of antibodies against HIV gp41 and gp160, (ii) reduced concentrations of antibodies to p17, p24, and reverse transcriptase (RT), and (iii) low or undetectable concentrations of anti-gp31. Those children on ART between 1 and 5 years old and with viral suppression (<400 RNA copies/mL) have higher levels of the preceding six antibodies than that seen in infants (52). These differences in antibody levels are likely to reflect the timing of ART on the one hand, but also the timing of the generation of HIV-specific-antibodies on the other, as suggested some time ago by Tomaras et al. (49). In other words, timing of HIV infection and persistence of antigen exposure may impact on the quantum of the HIV-1 specific antibody response in HIV-infected individuals (53).

Many past studies have described a corresponding decrease in anti-HIV antibody level with duration on ART, in participants with either primary or chronic infection as well as in perinatally infected children (48, 54–57). It thus appears that early ART initiation may interrupt HIV antigenic stimulation. In other words, ART may have the ability to sustain an HIV-specific antibody response when initiated early (58). Recently, Keating et al. (48), observed that (i) declining antibody levels during ART reflect lower levels of antigen production and/or viral replication and (ii) the higher levels of HIV-1-specific antibodies observed in individuals on suppressive therapy are associated with later initiation of ART, as well as cell-associated DNA and RNA levels. The suppressive activity of ART on the HIV life cycle thus seems to provoke a quasi-null HIV replication rate. However, anti-HIV antibody persistence during this period is probably stimulated by low levels of ongoing viral replication, or the production of translationally competent HIV-1 transcripts (59). To further illustrate this point, Brice et al., have shown that a significant proportion of virologically suppressed HIV-positive children stop producing antibodies, or have progressively lost HIV-specific antibodies, secondary to ART initiation before 2 years of age (56). McManus et al., have also demonstrated that ART limits HIV-specific antibody levels in the plasma of HIV-positive children. Thus, their antibody profiles become similar to those in HIV-uninfected children born to HIV-infected mothers (50). In the light of these details, it would be interesting to study the initial development of HIV-specific antibodies in early-treated patients, to determine precisely in what way the primary adaptative responses are affected, influenced, and restricted by ART. The decreases in specific antibody levels are, logically, likely to reflect a corresponding reduction in circulating antigenic HIV viral particles (56).

**Figure 2:**
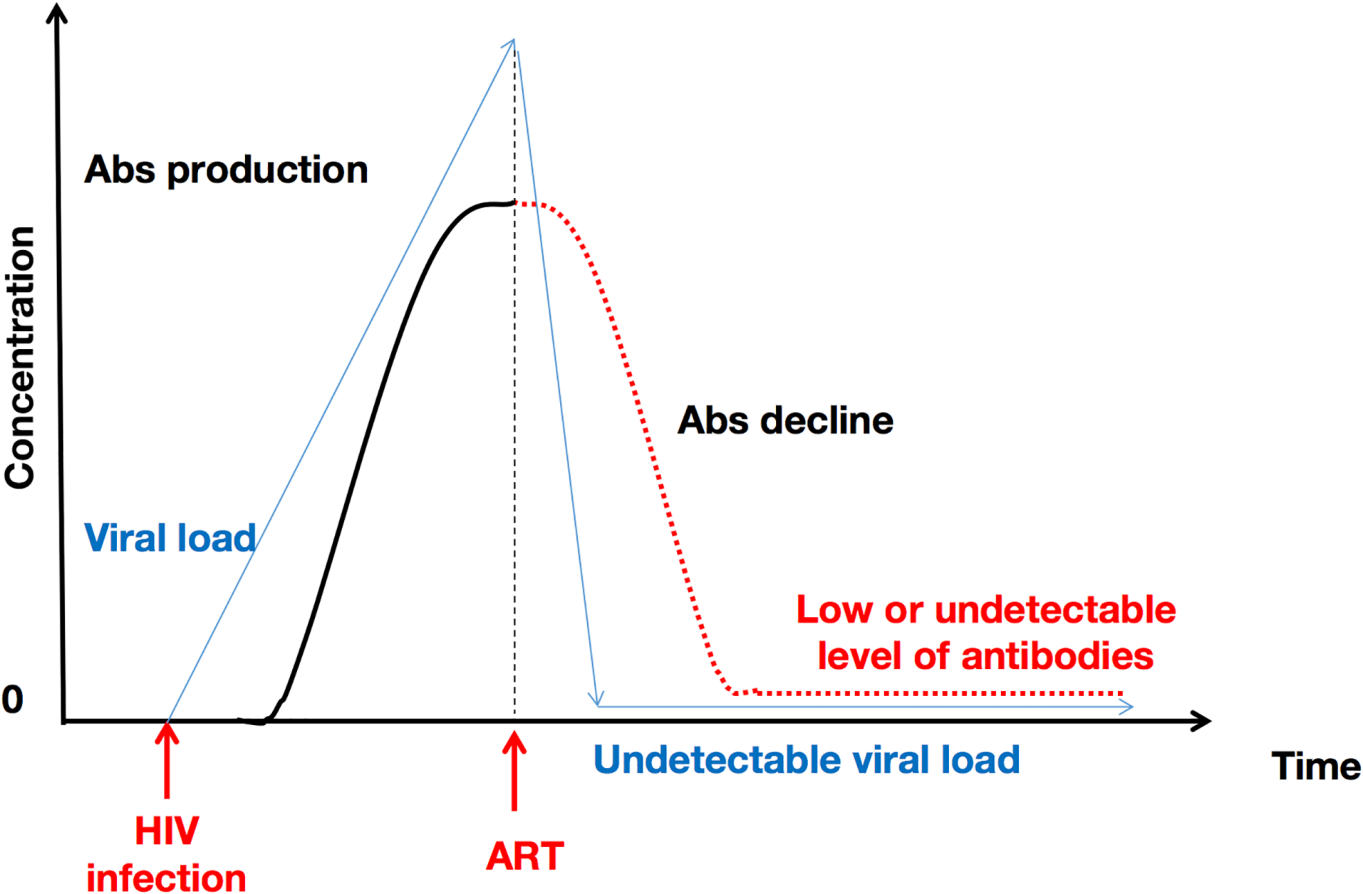
ART initiation and uninterrupted administration over time induces a decline in HIV antibody concentration. This represents the particular case of adults. ART provokes a decrease in viral RNA copies, which in turn explains the subsequent decrease in HIV-1-specific antibody levels. Abs, antibodies.

The nature of HIV infection in children and in adults is inherently too dissimilar to be able to compare them under identical parameters. Therefore, **Figure 2**. only presents the evolution of HIV-specific antibodies in adults who initiate and comply with ART treatment without interruption.

## Non-ART or Viral Load Factors Influencing HIV-1-Specific Antibody Production

Several factors may influence the production of HIV-1-specific antibodies. A comprehensive understanding of HIV antibody trends during HIV infection and the factors that may influence antibody expression is critical if one is to consider using antibody levels as a tool to reflect HIV-1 DNA levels.

### Age

In infancy, primary HIV infection is characterized by a high-level plasma viral load which decreases relatively little in the initial phases of the infection (60, 61). In adults, primary HIV infection is characterized by an initial viral load peak, followed rapidly by a 100–1,000-fold decrease in viral RNA copies, to reach a stable ‘set point’ within weeks (62). This age-associated difference noted in HIV viral kinetics may be explained (i) by the larger CD4+ T-cell compartment in infants and children (63–66), and (ii) a relatively less-robust innate immunity and/or a less-effective adaptative immune response in infants and children (66).

Age can influence the ontogeny of HIV-specific antibodies due to the differences observed in individual immune systems. As such, considering the antibodies that are passively transferred from the mother to the infant, researchers have found that baseline age correlates inversely with maternal antibody levels (50). Conversely, it is recognized that infants start producing their own antibodies after approximately 4 months (46). Compared with adults, some scientists suggest that the apparent delay in antibody production in infants, despite high levels of HIV replication, is due to their paucity of CD4+ T-cell-related influence (50). In other words, infant’s and children’s immune systems are characterized by an abundance of naive CD4+ T-cells, coupled with a limited capacity to generate antigen-specific memory cells (67).

### Biological Sex

Gender disparities in HIV pathophysiology remain a major area of concern as conflicting reports of more robust immune activation and antiviral responses in HIV-infected women have been published (as reviewed by Scully) (68). At the same time, it is worth noting that when using an identical assay (EIA-RI or recent infection enzyme immunoassay), at least two separate research teams have reported higher antibody levels in ART-treated women, compared with men (51, 54). Some studies have suggested that women are more likely to be categorized as spontaneous controllers of HIV (69, 70). It has been postulated that this gender-dependent HIV profile may be due to the activity of estrogen and estrogen receptor-1. Indeed, Das et al., have observed that estrogen and estrogen receptor-1 inhibit HIV transcription *in vitro* (71); however, further studies are required to elucidate the determinants of the “spontaneous controller” status of women.

### HIV Subtype

The role of HIV subtype in HIV-1-specific antibody production remains to be clarified. It is known that subtype AE is more transcriptionally active and produces a lower degree of latency than subtype B, due, in part, to the GA-binding protein (GABP) site present in the subtype AE long terminal repeat (LTR) (72). Furthermore, researchers have shown that subtype C is less functional and more sensitive to apolipoprotein B mRNA editing enzyme catalytic subunit 3G (APOBEC3G)-mediated viral inhibition, compared to subtype B or subtype AG (73). The subtype C viral genome was observed to be hypermutated with APOBEC3G, with a limited ability of subtype C to infect cells, and thus to replicate. Subtype D has been shown to be more inclined to induce latency than other subtypes in an *in vitro* model of latency (74). Bachmann et al., found that decay of the latent reservoir was significantly faster in non-B individuals, compared to subtype B-infected individuals (75). Furthermore, it was found that subtype C-infected individuals had lower total levels of HIV-1 DNA than subtype B-infected individuals. In the light of such details, it is legitimate to hypothesize that a less virulent strain (that is more inclined to latency) allows the immune system to develop a stronger humoral antibody response than virulent stains which are highly active with respect to viral replication. This hypothesis is purely speculative, and further targeted investigation could help to determine the antibody profiles in individuals infected by different HIV subtypes. For now, it has only been established that more virulent strains induce a greater depletion of CD4+ T-cells (76) and poorer immune recovery (77).

### Co-Infections

Despite being on ART, HIV-infected individuals are known to exhibit an underlying chronic inflammatory state. HIV-1 related-immunodeficiency increases susceptibility to several pathogenic viruses, including cytomegalovirus, the hepatitis viruses, and other viruses, which also contribute to the chronic inflammation (78). Currently, despite some exceptions (79), most studies suggest that HIV reservoir size is shaped in a complex manner by the prevailing inflammatory environment. Simply stated, comorbid infection further enhances existing inflammation, which provokes further immune activation, thus resulting in higher reservoir sizes (19, 80, 81). Similarly, several researchers have found that HIV/simian immunodeficiency virus (SIV) DNA is enriched in cells that express immune activation (82–85). It has also been shown that inflammation of lymphoid tissue is mainly responsible for the immune dysfunction and the reduced immunity to HIV-1. Thus, the question arises as to what the implication of co-infections would be on the dynamic evolution or production of HIV-1-specific antibodies. Perhaps, antibody production in co-infected individuals may be reduced, slower, or dysfunctional. Further investigation into this area of inquiry is warranted.

### Allogeneic Stem Cell Transplantation (Allo-HSCT)

Many studies have reported a diminution or clearance of HIV-specific antibodies after allo-HSCT. Indeed, a clinical trial by Salgado et al., including 6 participants with viral suppression resulting from uninterrupted administration of cART, and who survived more than 2 years after allo-HSCT (with CCR5 wild-type cells), reported that all the patients had lost their anti-gp18 antibody, while some displayed decreasing p31 antibody levels (2 participants), or absent anti-p55 and anti-p24 antibodies (2 participants). These observations brought the authors to suppose that a longer interval after allo-HSCT seemed to be associated with greater antibody clearance among patients receiving cART (86). Similarly, Koelsch et al. (87), and Henrich et al. (88), present evidence that implies that allo-HSCT may modify the serological evolution of HIV-positive individuals. In the study by Koelsch et al., four cases were considered in which 3 patients (designated as patients A, B, and C) highlight the substantial changes to the HIV immune response in patients undergoing allo-HSCT. Briefly, the antibody concentrations in patient A were very low at the first measured time point (4 years post-transplant), but increased after the patient experienced viral rebound at the second measurement (31 weeks later). In patient B, antibody concentrations were measured twice, once at 3 years and once at 7.5 months post transplantation. At both time points, antibody concentrations were extremely low. In patient C, a progressive diminution of antibody concentrations was reported between months 6 and 12 following transplantation (87). Henrich et al., have also demonstrated that antibodies levels declined (in two patients, A and B) during the period of virologic suppression post-HSCT (>1000 days). They also noted that an antiretroviral treatment interruption (ATI) was followed by a viral rebound associated with antibody level increase. The well-known cases of the “Berlin” (89) and “London” (90, 91) patients are also worth mentioning. Officially known as functionally cured of HIV infection, both received allogeneic bone marrow transplants from a naturally-mutated CCR5 gene (CCR5 delta 32) donor. Reports suggest that the HIV antibody levels of these patients were readily detectable. Although their HIV antibody levels have continued to decline over time, the levels remained above the cut-off levels for HIV negative individuals (89–91).

## Relationship Between HIV-1-Specific Antibodies and HIV-1 DNA

Several published articles suggest that measurement of HIV-specific antibodies may provide a reliable, cost-effective, and highly reproducible tool to estimate HIV reservoir size in HIV-positive individuals on ART. This concept was conceived after researchers observed lower residual blood cell-associated HIV-1 DNA levels (58, 92–96) and low or absent HIV-1-specific antibody levels (66, 97, 98) in the first few months of life in perinatally infected infants, when viral suppression occurs following ART initiation. However, there remains no consensus regarding this point, as some research teams failed to observe any significant association between these two variables (55, 57). Although the individual methods of investigation vary (**Table 1**), the observations related to this issue are worthy of intellectual contemplation.

**Table 1:** Summary of published articles (2016-2021) investigating HIV-1-specific antibody capacity to estimate HIV-1 reservoir size.

Study participants		Methods of investigation		HIV Abs can predict HIV reservoir size		Reference
Category of participants	Sample size	Technique(s) and/or immunoassay(s)	HIV-specific antibody (ies)	Yes, No, Maybe	Antibody (s)	
Adults	51	LIPS	gp120, gp41, CA, MA, RT, INT, PR	Maybe	gp120, INT, PR	(55)
	274	Vitros HIV 1 + 2 assays avidity enzyme immunoassay	P24, gp41,	Yes	P24, gp41	(48)
	683	Enzyme immunoassay (EIA)	gp41	Maybe	gp41	(51)
	78	Fluorescently coded microspheres conjugated with HIV antigens of interest	gp41, gp120, p51, gp140	Yes	gp41, gp120	(99)
Children	69	Western blot	Gp160, gp120, P66, P55, P51, gp41, P39, p31, P24, P17	Yes	gp120, gp41, p31, P24	(34)
	97	The fourth-generation ARCHITECT HIV Ag/Ab Combo assay	gp41	Maybe	gp41	(56)
	61	ELISA	gp160, gp41,p24, p17, RT, p31	Yes	gp160, gp41	(52)
	46	ELISA	gp160, gp41, P24, P17, P31, RT	Yes	gp160, gp41	(50)

In 2016, Lee et al., found (i) a significant positive correlation between anti-HIV integrase and HIV RNA in gut-associated lymphoid tissue and (ii) a negative correlation between anti-HIV matrix and integrated HIV DNA in CD4+ T-cells. Of note, a 0.35-fold decrease in the anti-HIV matrix count was associated with a 2-fold DNA increase. However, the application of more stringent statistical analysis (the Bonferroni-adjustment) has revealed that these associations were not actually statistically significant. Notwithstanding this, Lee and his colleagues have suggested that antibodies against enzymes involved in HIV replication might be of better use than those against HIV structural protein. The level of antibodies against HIV p24, matrix, and gp 41 has been demonstrated to likely reflect the level of HIV replication in an individual (100).

In 2017, Brice et al., observed that the low activity of anti-gp41 was associated with low levels of anti-HIV antibodies and a younger age of ART initiation. The study population comprised of vertically-infected HIV-positive children (median age of 3.3 years, receiving ART for a median 5.4 years) with virological suppression. An interesting finding was that the overall analysis revealed that no correlation existed between anti-gp41 antibodies and HIV DNA. However, children who initiated ART before the age of 2 years had a very low HIV reservoir, in addition to having low antibody levels and activity (56).

In late 2018–early 2019, a French team reported that anti-gp41 levels reflect HIV cellular reservoir size in long-term ART-treated adults (median age 41 years old, treated for a median of 10.7 years, and 77.5% were men). Actually, Delagreverie et al., have demonstrated that a lower titer of anti-gp41 correlates with male gender, longer viral suppression, and lower HIV-1 DNA burden (anti-gp41 and cell associated HIV-1 DNA quantification). Of note, (i) anti-gp41 levels does not correlate with the overall duration since HIV diagnosis, (ii) lower titers of anti-gp41 were observed in participants with non-detectable HIV-1 DNA compared to those with quantifiable HIV-1 DNA, and (iii) anti-gp41 levels were higher in women than in men (51). The results of Delagreverie et al’s., study thus concur with that of Keating et al., from 2017 (in which the study population was recently-infected individuals), despite different approaches in terms of analysis (EIA-RI versus gp41 limiting antigen avidity assay). Indeed, Keating et al., have observed a correlation between anti-gp41 levels and PBMC-associated HIV-1 DNA for up to 6 years of viral suppression (48). Interestingly, Delagreverie et al., and Brice et al., used the EIA-RI approach to estimate anti-gp41 levels; however, lower overall numbers were observed by Brice and his colleagues. This may be explained by the current use of dried serum spot samples and/or the differences in immune response between children and adults, as the study of Brice et al., only included infants. This suggests that antibody titers may be artifactually underestimated if an appropriate sampling method is not used for sample collection.

In 2019, scientists from the University of Massachusetts Medical School have demonstrated that quantitative HIV antibodies correlate with cell-associated DNA levels in children on ART (50). In contrast to the investigation of Brice et al., who did not include children who started ART before 5 months of life, MacManus and her colleagues (50) have included children stratified by age at ART initiation, as early therapy (<3 months of life), and late therapy (3 months to 2 years of age). Furthermore, MacManus et al., have included HIV-negative children born to HIV-positive mothers as controls, and instead of measuring only HIV anti-gp41, they measured antibodies against HIV-1 (i) envelope (gp160, gp41), (ii) gag (capsid, p24, matrix, p17), (iii) reverse transcriptase (p66, p55), and (iv) integrase. Overall, they found that the diminution of the measured HIV-specific antibodies (other than anti-p17) in the early therapy group (and virologic response group) was similar to that of the control group. Most importantly, they found that after one year of age, the levels of antibodies such as anti-gp160 and anti-gp41 were directly associated with circulating HIV DNA levels (R^2 =^ 0.37; p<0.05). Logistic regression has estimated that each unit increase in log anti-gp160 was associated with a 6-fold increase in HIV DNA level. Additionally, they also observed that after one year of age, levels of anti-p31 and anti-p17 are directly associated with plasma HIV RNA levels (R^2 =^ 0.59; p<0.05) (50).

In late 2019–early 2020, McManus, in a related further publication (52), indicated that quantitative anti-gp41 and anti-gp160 levels may serve as rapid and inexpensive tools to screen for low HIV DNA levels in PBMC’s in children with viral suppression. Indeed, two approaches, namely, the receiver operator curve (ROC) approach and the ensemble learning approach, have identified anti-gp41 and anti-gp160 as important predictors of HIV-1 DNA when HIV DNA counts are estimated at less than 10 or less than 100 copies per million PBMC. In 2020, another research team characterized humoral biomarkers of reservoir size in controllers and chronic progressors. Indeed, Das et al., have provided insightful information with regards to whether antibody profiles tracked consistently with estimates of reservoir size across elite controllers, viremic controllers, and untreated chronically infected progressive patients not on ART. After demonstrating that antibodies can indeed be used to discriminate between individuals with higher and lower HIV DNA levels, they have further identified specific antibodies (IgG-gp41, r=0.489, *p*<0.001 and IgG2-gp41, r=-0.399, *p*=0.006) which differentiated these controllers by reservoir size. Das et al., further investigated whether humoral biomarkers of reservoir size can estimate the reservoir size in ART-treated subjects, and they observed that IgG-gp41 (r=0.597, *p*=0.011) and IgG3-gp120 (r=0.135, *p*=0.029) HIV-specific antibodies correlate with the size of the HIV reservoir in these subjects (99).

In the aforementioned studies, the participants were under ART and had undetectable HIV viral loads, were from the same patient category (either children or adults), and had no co-infection to influence HIV-1-specific antibody production, and to also ensure the comparability of the results. Overall, it appears that these studies favor utilizing anti-gp41 to determine HIV-1 reservoir size. However, only studies conducted in children show a global trend in favor of using HIV-specific antibodies, notably anti-gp41, to monitor HIV reservoir size. In contrast, in studies in adults, approximately half remained skeptical of the use of HIV-specific antibodies to monitor HIV reservoir size. Knowing that the nature and manifestation of HIV infection is vastly different in children compared to adults, it would be wise to wait for additional investigational evidence to make firm conclusions regarding the possibility of using HIV-specific antibodies to monitor HIV DNA in adults. Moreover, ELISA and enzyme immunoassays seem to be the most commonly used methods to investigate the relationship between HIV-1-specific antibodies and HIV-1 DNA in children and adults, respectively. This highlights the presence of additional challenges potentially affecting the relationship between HIV-1-specific antibodies and HIV-1 DNA.

## Additional Challenges

Based on contemporary literature, we note that the analysis of the relationships between HIV-1-specific antibodies and HIV-1 DNA may be influenced by the specific methods of investigation. Indeed, it is critical to state which particular method should be used to measure anti-gp41; the specific method used could, otherwise, induce difficulties and/or differences in terms of result interpretation. This is a matter of particular concern in the case of the Berlin patient, for example, where the antibody level measured at four post-transplantation time points with the undiluted HIV-1/2 VITROS assay was relatively stable (~75-80 signal/cut-off ratio) at all four post-transplantation time points considered. However, the same objective using different methods, such as the detuned HIV-1 VITROS assay (from ~0.38-0.39 signal/cut-off ratio to ~0.30 signal/cut-off ratio at the end of the study), or the limiting antigen avidity assays (from ~0.31 normalized optical density to ~0.27 normalized optical density at the end of the study), indicated lower HIV antibody levels, which tended to decrease over the time (89). Similar observations were made by investigators studying the London patient (90). Therefore, further investigation aimed at establishing consensus with respect to the best methods to be used for such tasks, is warranted. Similarly, the best and most appropriate laboratory methods for use in adults and children should be also investigated.

Considering ART-treated patients undergoing HIV curative strategies and in need of HIV DNA estimation *via* HIV specific antibody quantification, viral rebounds subsequent to ART discontinuation or the emergence of treatment resistant strains should be carefully monitored. The results by Koelsch et al. (87), and Henrich et al. (88), show that viral rebound even after allo-HSCT results in an antibody level increase on the one hand, but may also favor the augmentation of HIV reservoir size on the other. If HIV reservoir size reaches more than 100 copies per million PBMC, the currently understood relationship between HIV-specific antibodies and HIV DNA, as outlined by McManus et al. (52), will no longer be applicable.

Finally, we believe that sample collection and preservation before laboratory studies are conducted, should be particularly taken heed of. As suggested by comparing the study by Brice et al. (56), to the investigation by Delagreverie et al. (51),, dried serum spot samples do not seem appropriate, as they tend to produce relatively lower titers of HIV-specific antibody results. A standardized process for sample collection is, therefore, necessary. In addition, sample size represents an interesting marker of precision. Conclusions based on a large sample are known to provide a much more realistic result compared to those from a limited sample. From the studies cited in this article, the screening of more than five hundred individuals (352 adults and 176 children) seems to favor the notion that gp41 may be a realistic option for HIV-1 DNA quantification in HIV-infected individuals. However, further investigation using larger sample sizes are necessary to generalize results to the large population of people living with HIV globally [officially, 37.6 million in 2020 (2)].

## Conclusion

Contemporary literature demonstrates a trend in favor of using HIV-1-specific antibodies to predict HIV-1 reservoir size [5 of 8 papers (62.5%), **Table 1**], particularly in HIV-positive children [3 of 4 papers (75%), **Table 1**]. With respect to adults, it is currently not possible to make a similar conclusion, as we failed to find a trend in the data in favor of such an approach. Interestingly, anti-gp41 (one of the first antibodies to be produced in infants and adults) is likely to be the most promising antibody candidate for use as a potential marker for this task. Although HIV-1-specific antibody use seems to be a simpler, less expensive, and less restrictive laboratory tool, their absolute levels are sensitive to several determinants, including viral load, ART use, biological sex, age, HIV subtype, and presence of co-infections. In light of this, we believe that further investigation is warranted in order to provide clearer and definitive recommendations regarding estimation of HIV-reservoir size, particularly in adults.

## Author Contributions

SZ and FS wrote the first draft of the manuscript. YC provided critical revision of the manuscript. All authors contributed to the article and approved the submitted version.

## Funding

This work was supported by Chongqing Public Health Medical Center Youth Innovation Program (2019QNKYXM04) and the Joint Medical Research Projects of Chongqing Municipal Health Commission and Chongqing Municipal Science and Technology Bureau (2020GDRC004, 2020MSXM097, 2020FYYX066).

## Conflict of Interest

The authors declare that the research was conducted in the absence of any commercial or financial relationships that could be construed as a potential conflict of interest.

## Publisher’s Note

All claims expressed in this article are solely those of the authors and do not necessarily represent those of their affiliated organizations, or those of the publisher, the editors and the reviewers. Any product that may be evaluated in this article, or claim that may be made by its manufacturer, is not guaranteed or endorsed by the publisher.
